# Do Individuals Perceive Income Tax Rates Correctly?

**DOI:** 10.1177/1091142115615670

**Published:** 2017-01-01

**Authors:** Michael Gideon

**Affiliations:** 1 Economist, Washington, DC, USA

**Keywords:** average tax rates, marginal tax rates, tax perceptions

## Abstract

This article uses data from survey questions fielded on the 2011 wave of the Cognitive Economics Study to uncover systematic errors in perceptions of income tax rates. First, when asked about the marginal tax rates (MTRs) for households in the top tax bracket, respondents underestimate the top MTR on wages and salary income, overestimate the MTR on dividend income, and therefore significantly underestimate the currently tax-advantaged status of dividend income. Second, when analyzing the relationship between respondents' self-reported average tax rates (ATRs) and MTRs, many people do not understand the progressive nature of the federal income tax system. Third, when comparing self-reported tax rates with those computed from self-reported income, respondents systematically overestimate their ATR while reported MTR are accurate at the mean, the responses are consistent with underestimation of tax schedule progressivity.

Every man is “aware” of taxes, especially in this year of 1963. The extent of this awareness has rarely been examined, despite the ever-increasing importance of the public sector. Given our ignorance about tax awareness or tax consciousness, it is surprising that some economists are so willing to predict the effects of changes in the tax structure on individual behavior. If we do not know people's tax consciousness, how can we know the extent to which changes in their tax burden will affect their behavior? Enrick (1963)

Taxes and subsidies distort incentives, yet a substantial body of research finds only modest behavioral responses to tax changes. To understand these results, recent work has focused on whether various “optimization frictions” such as inattention, switching costs, and inertia dampen responsiveness. However, the explanation could be even more fundamental—that people have heterogeneous and potentially biased perceptions of tax incentives.^1^

Enrick (1963) recognized half a century ago that people might respond to changes to their perceptions of taxes rather than to changes in the tax structure itself. Using similar logic, Sheffrin (1994) makes the case that knowledge of the tax system shapes public opinion, which influences the formation of tax policy. Yet without measuring individual-level perceptions of tax rates, we cannot estimate how behavior reacts to (perceived) policy changes and how perceptions influence policy formation.

Despite its theoretical and practical importance, little attempt has been made to directly measure and systematically analyze income tax rate perceptions among US taxpayers. Among the studies looking at awareness of income tax liability (e.g., see, Enrick 1963, 1964; Wagstaff 1965; Auld 1979; Gemmell, Morrissey, and Pinar 2004) or marginal income tax rates (MTRs; e.g., see, Break 1957; Gensemer, Lean, and Neenan 1965; Brown 1968; Lewis 1978; Fujii and Hawley 1988) most were years ago, outside of the Unites States and without detailed enough data needed to characterize perceptions of both tax liability and MTR.

In this article, I describe the results of an exploratory study of income tax perceptions. Data come from a battery of questions from the 2011 wave of the Cognitive Economics Study (CogEcon), which was fielded between October 2011 and January 2012. Specifically, the survey questions aim to measure the respondents' own average and marginal tax rates (MTRs), their perceptions of top MTRs and their expectations of future MTRs. There is compelling evidence of systematic errors in how people perceive US income tax rates.

After describing the survey instrument, I discuss three sets of findings. First, I analyze knowledge of MTRs for households in the top tax bracket. I find convincing evidence that respondents underestimate the top MTR on wages and salary income, overestimate the MTR on dividend income, and underestimate the currently tax-advantaged status of dividend income.

Next, I analyze perceptions of respondents' own self-reported average tax rate (ATR) and marginal tax rate. I find that many people do not understand the key aspects of the federal income tax system, even parameters economists typically assume influence behavior. Respondents are much more likely to answer the question about own ATR than questions about MTRs. Among those who provided both ATR and MTR, with at least one nonzero, only a third of respondents correctly reported an MTR larger than their reported ATR.

Last, I compare perceived ATR and MTR with rates computed from self-reported income. I present evidence that respondents systematically overestimate their ATR. While the reported MTRs are accurate at the mean, respondents with lower income overestimate MTR and those with higher income underestimate MTR. This pattern is consistent with underestimation of tax schedule progressivity.

The rest of the article proceeds as follows. In the next section, I describe the survey instrument. In the following section, I present the results and I conclude in the final section by relating these findings to previous studies on tax perceptions and discussing implications for research and policy.

## Survey Methodology

Data measuring tax rate perceptions come from the 2011 wave of the CogE-con.^2^ I helped develop new questions about federal income tax rates for the CogEcon 2011 survey instrument, which was fielded between October 2011 and January 2012. The exact wording and ordering of the five questions are in the Appendix.

The introduction to this set of questions includes a definition of MTRs and explicitly states that respondents should not include state or local taxes or payroll taxes for social security and medicare. After this introduction, there are two questions about MTRs imposed on households in the top tax bracket. First, respondents are asked what they think is the marginal tax rate on wage and salary income for these top bracket households in 2010 and the marginal tax rate on dividend income for these households in 2010. This question elicits a measure of tax rate knowledge. In tax year 2010 the highest statutory marginal tax rate on wages and salary was 35 percent, while the highest rate on dividend income was 15 percent. The second question about top MTRs elicits expectations about the future. It repeats the first question but asks respondents for rates they expect to be in place in 2014. These questions are followed by three questions about the respondents' own tax rates. The first is about their ATR in 2010, the second about their marginal tax rate in 2010, and the last about their expected marginal tax rate in 2014.

The questions are written in precise yet simple language to elicit perceptions of average and marginal tax rates. The question refers to an “income tax bracket” as a way to elicit perceptions of one's statutory marginal income tax rate without explicitly distinguishing between statutory and effective rates. The effective marginal tax rate would take into account how the value of deductions and credits sometimes depends on income; a one-hundred dollar increase in income might lead to a larger than one-hundred dollar increase in taxable income (TI) and the effective tax rate would be larger than the statutory rate. While respondents might be confused about whether the question is asking about statutory or effective rates, the people who understand the difference are expected to lean toward giving the statutory rate.^3^

For the ATR question, CogEcon intentionally uses a clearly specified tax concept but a vague definition of income. This is because CogEcon measures household income using a broad question earlier in the survey. If the survey specified adjusted gross income (AGI), then knowledgeable respondents would use this income concept while others would not, and there would be no way to disentangle knowledge of the tax concept from knowledge of the income concept. Finally, CogEcon asks for the ATR before asking about marginal tax rate to get an indicator of whether respondents understand that in the progressive U.S. tax system, the ATR is always (weakly) less than the marginal tax rate.

## Results

The sample consists of 748 respondents who completed CogEcon 2011. The sample consists of older households (mean 67.9 years and median 66.2 years) with slightly higher income (mean $83,600 and median $65,600). Roughly two-thirds of these respondents are married, 57 percent are female, and 50 percent are working, with mean education of over fourteen years. Table 1 compares characteristics of the sample with the U.S. adult population, using data from the U.S. Census Bureau. In addition to being older than the general population, the CogEcon sample is higher income, more highly educated, and more likely to be homeowners. Because the CogEcon sample is older than the general population, it is not surprising that there is a larger share of women and are less likely to be working.

**Figure 1:**
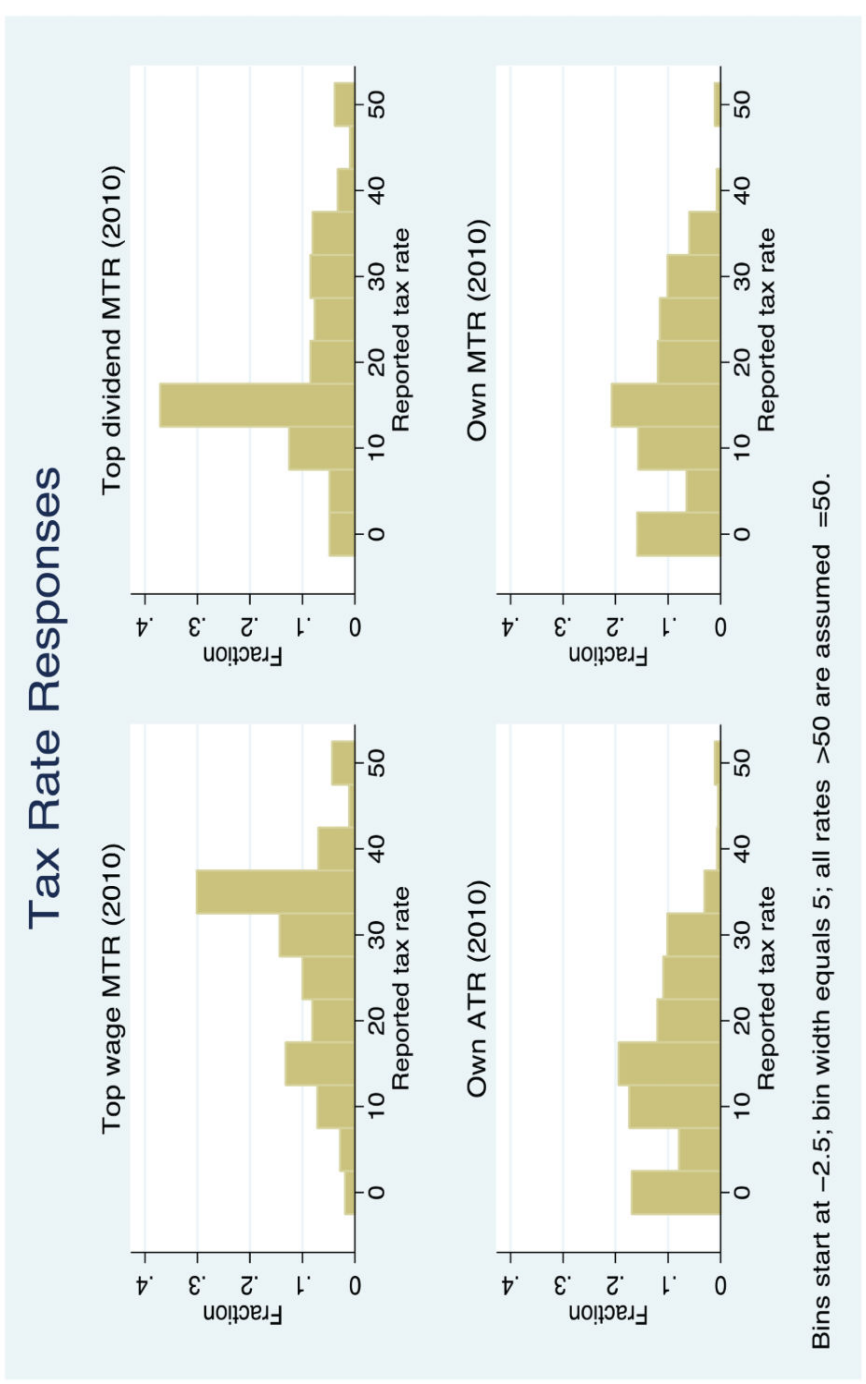
Histograms of responses about income tax rates. *Source:* CogEcon 2011.

The main findings come from the descriptive patterns in the data. Table 2 provides a general summary of responses to the five questions, with the mean, standard deviation, quartiles, number of observations, and the frequency of missing data. Figure 1 provides a visual summary of tax rate responses using a histogram for each of the questions about 2010 (current) tax rates. There are concentrated responses at 0, 15, and other statutory MTRs or round numbers.

## Knowledge of Top Income Tax Rates

Looking at the summary statistics in table 2 the perceived top marginal tax rate on wage/salary income has a mean of 27.4 percent and a median of 30 percent, while the perceived top marginal tax rate on dividend income has a mean of 20.0 percent and a median of 15 percent. Since the correct answers are 35 and 15 percent, respectively, this implies respondents underestimate the MTR on wage/salary income and overestimate the MTR on dividend income. These results combined suggest that people significantly underestimate the currently tax-advantaged status of dividend income.

While the modal rates are correct, only a small fraction report these rates. Overall, 131 respondents correctly reported 35 percent as the top rate on wage and salary income. This means that only 17.5 percent of all respondents reported the true rate, and only 24.7 percent of those who provided an answer to this question (531 observations) reported the true rate. As for the top rate on dividend income, 185 correctly reported 15 percent which is 24.7 percent of all respondents and 35.6 percent of those who provided an answer to this question (520 observations). Overall, only 57 respondents reported the correct answer for both, which is only 7.6 percent of all respondents and 11.0 percent of those who provided an answer to both questions (518 observations).

Even if respondents did not know the exact tax rates faced by high-income earners, they may still know that dividend income is taxed at a lower rate. Table 3 summarizes the responses about the top MTR on wage and salary income and the top MTR on dividend income. First, roughly 30 percent of respondents skipped one question or the other; of those, most skipped both. Second, among respondents who answered both questions, 297 correctly reported that the rate on wage and salary income is higher than the rate on dividend income. This was 39.7 of the sample overall, and 57.3 percent of those who answered both questions (518 observations), which suggests that many people did not even know dividends are tax-advantaged.

My interpretation of the data requires a few caveats. First, many sources of investment income are taxed as ordinary income, as is the case with capital gains. Respondents who know the top rate on wage/salary income but not the intricacies of capital taxation might report 35 percent for both. Second, because 15 percent was mentioned in the example used to explain the definition of marginal tax rate, it could have anchored respondents when they did not know the answer. So, respondents with little knowledge of taxes might show up in the data as correctly reporting the tax rate on dividends yet underestimating their tax-advantaged status. Finally, for this older adult sample, dividends were taxed as ordinary income for much of their lives.^4^ If perceptions of tax rates change slowly, then people may still think dividends are taxed the same as wage/salary income. In this case, misper-ceptions about the tax-advantaged status of qualified dividends should be interpreted as evidence that beliefs have not yet been updated rather than evidence of systematic errors.

## Comparing Own ATR and MTR

Looking again at table 2, self-reported ATR has a mean of 15.4 percent, and self-reported marginal tax rate has a mean of 16.1 percent. For both questions, the median is 15 percent. Respondents think tax rates (both their own and the top rates) are going to be higher in 2014 than they were in 2010.

**Figure 2:**
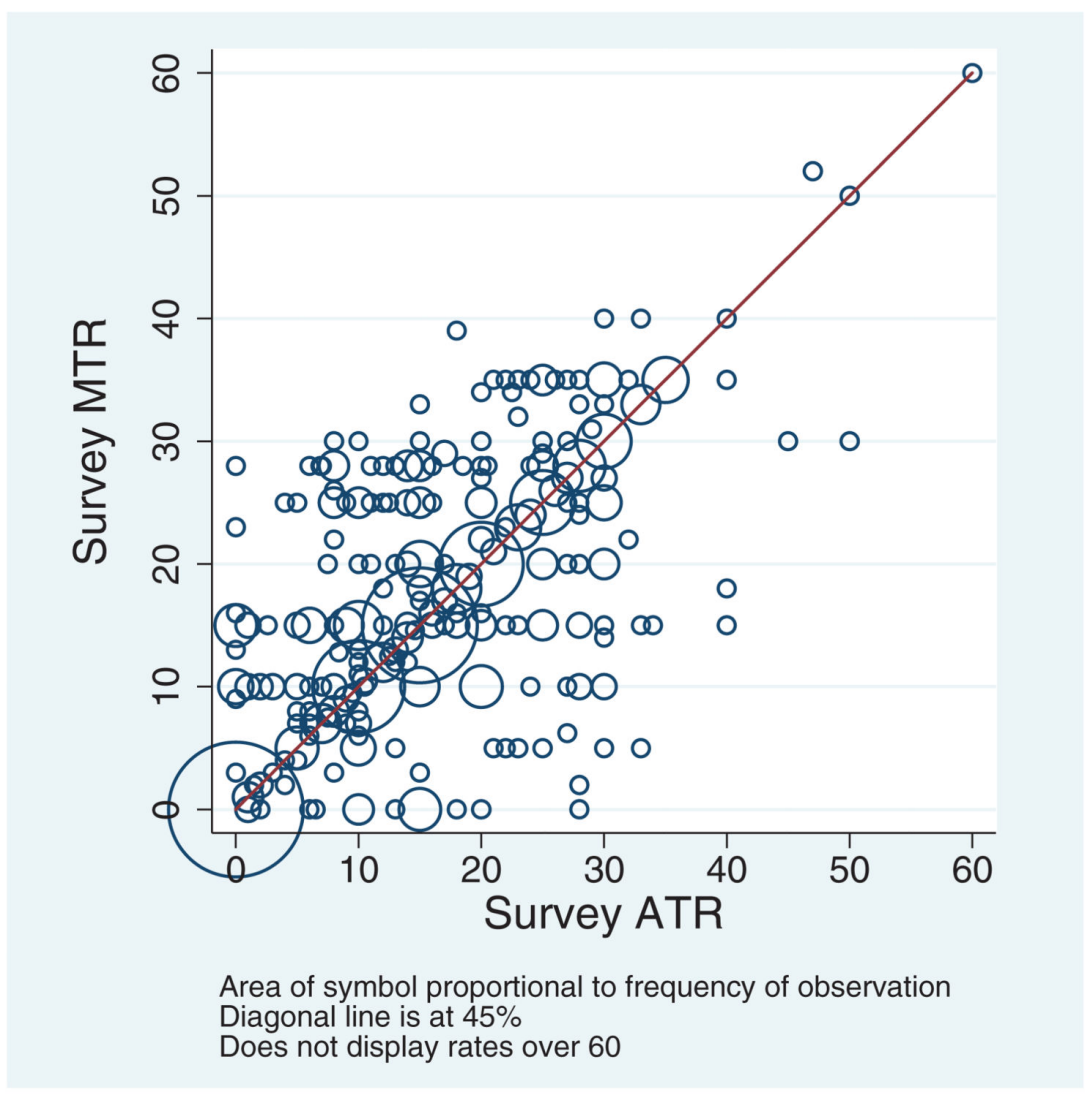
Scatterplot of survey average tax rate (ATR) and marginal tax rate (MTR) measures (2010; weighted). Source: CogEcon 2011. *Note*: The figure shows a scatterplot of MTR and ATR survey responses. Because some respondents reported the same pair of tax rates, the size of the circle reflects the frequency of the pair.

Figure 2 displays a scatterplot of MTR and ATR survey responses, where the size of the circle reflects the frequency of that pair. The scatterplot of tax rate responses shows variation in the empirical distribution of MTR and ATR. While many respondents reported 10, 15, 20, and 25 percent for both MTR and ATR, there is substantial dispersion among those who did not give the same number for both.

To see this result another way, table 4 presents the distribution of respondents' answers across categories of average and MTRs. The first three rows are the percentage of respondents (of the subsample) who skipped both questions, the percent who only skipped the MTR question but answered the ATR question and the percent who answered zero for both. The next three rows show the percentage of respondents with survey MTR larger than, smaller than, and equal to ATR, conditional on answering both with at least one nonzero numbers. table 4 shows this for the entire sample of 748 respondents and also the 679 (90.8 percent) who filed a tax return for 2010.

Roughly, 15 percent of respondents skipped both questions and another 10 percent answered the question about ATR but not about MTR. Only one respondent skipped the question about the ATR and answered the question about their marginal tax rate. My interpretation of the different response rates is that people skipped the question when they did not know the answer and fewer people knew the answer about MTR than about ATR. People seem to have an intuitive sense of how much they pay in taxes overall but do not understand the tax structure enough to have a sense of how this amount changes when their income changes. The reason for this interpretation is that people who claimed to understand the tax system were more likely to answer the MTR question. Nevertheless, it is difficult to disentangle whether people were confused about the question itself or understood the question but did not know the answer. Regardless, this is a surprising result because the standard approach to empirical tax analysis focuses on marginal rather than average rates because of their incentive effects.

Comparing self-reported average and marginal rates provides an indicator of whether respondents understand that in the progressive tax system the ATR is always (weakly) less than the marginal tax rate. The relationship between MTR and ATR is due to the facts that statutory MTRs increase with the level of TI and that deductions and exemptions are always strictly positive. Whenever TI is positive, the second fact implies that MTR is strictly greater than ATR. Overall, only 22.2 percent of respondents reported MTR strictly greater than ATR. Among the subsample of respondents who answered both questions (with at least one nonzero number), 33.7 percent of respondents reported the correct relationship (or 166 of 492).

The largest share of respondents gave the same number for both ATR and MTR or 37 percent of the sample and 29 percent if those who reported zero for both are excluded. One explanation for this pattern is that reported tax rates are rounded answers, in which case we cannot conclude that people think the marginal and average rates are the same. However, it is also reasonable to infer that many people think the two rates are the same. This is consistent with previous evidence from de Bartolome (1995), who shows that in an experimental setting people typically use their ATR in place of their MTR. Finally, as described in Liebman and Zeckhauser (2004), people might think that all income is taxed at the marginal tax rate. This suggests that people are reporting their perceived MTR when answering the questions about ATR and MTR.

One way to address this question is to look at whether people are reporting a number that could be a statutory tax rate. Table 5 shows the fraction of respondents who reported a statutory MTR, broken down into categories based on responses to ATR and MTR. Overall, approximately half of the respondents reported a marginal tax rate equal to a statutory MTR rate. Among those who reported MTR < ATR, only 42 percent said a statutory rate. The fraction increases to almost 50 percent for the group who reported MTR = ATR and 67 percent for those who correctly reported MTR > ATR. This suggests that some people know about the tax rate schedule and answer based on where they think they are in the tax schedule. It also provides strong evidence that not everyone knows the statutory marginal tax rate schedule. Finally, it suggests that some of the respondents who report MTR = ATR may in fact be thinking in terms of marginal rather than ATRs.

## Comparing Self-reported and Computed Tax Rates

Without administrative data, I cannot directly compare self-reported rates and their true values. Instead, in order to evaluate the magnitude of these responses, I compare self-reported ATR and MTR with tax rates computed from self-reported income.

Self-reported income data collected in CogEcon 2011 are used to compute TI. First, I use the NBER TAXSIM tax rate calculator to transform this vector of income variables into AGI; see Feenberg and Coutts (1993) for a discussion of TAXSIM. Then, I compute TI from AGI by subtracting exemptions and deductions. Information about dependent exemptions was collected in the survey, and I assume that all taxpayers claim the standard deduction. See Gideon (2014) for details about the income variables and the construction of AGI and TI.

TI and tax return filing status determine statutory MTR and tax liability, and the computed ATR equals tax liability divided by AGI. Single respondents are assumed to file as single (rather than head of household), and married respondents are assumed to file jointly.^5^ I assume the true marginal tax rate is the statutory rate, which is consistent with how the question was worded.^6^ Statutory MTRs for wage and salary income were 10, 15, 25, 28, 33, and 35 percent, the levels set in the Jobs and Growth Tax Relief Reconciliation Act of 2003. The income thresholds depend on filing status (married or single). Table 6 presents the TI thresholds associated with these MTRs for tax year 2010, broken down by filing status.

**Figure 3:**
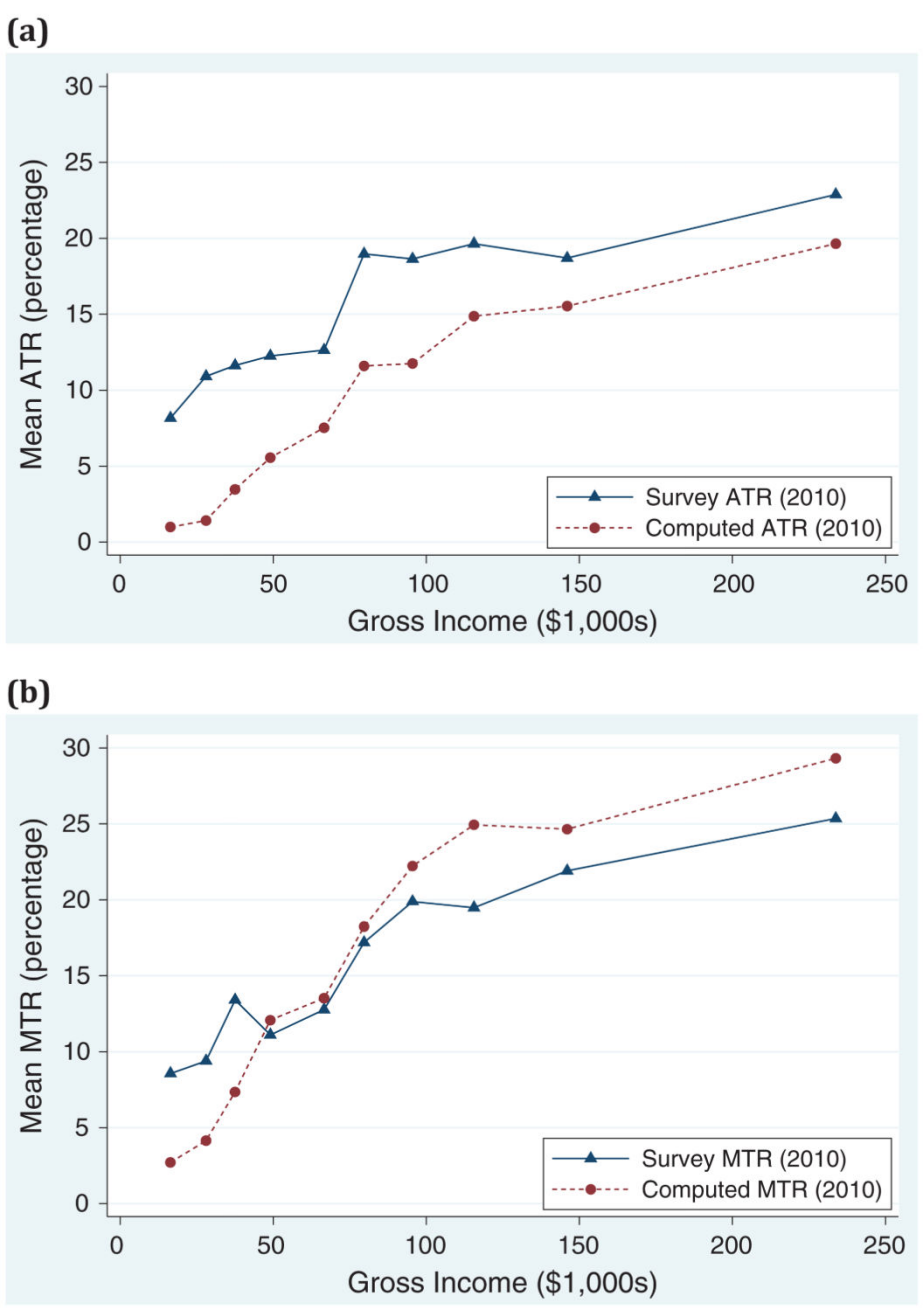
Binned means of survey and computed marginal tax rate (MTR) and average tax rate (ATR), across gross income (2010). (a) Average tax rates. (b) Marginal tax rates. *Source:* CogEcon 2011. Note: Observations are grouped into ten equally sized bins of income. The mean tax rate is graphed at the mean income within the bin. The sample used for these figures was restricted to people who provided both ATR and MTR, such that the sample is the same in both figures. Respondents with income above $500,000 are removed to limit the mean of the binned income for the top bin.

Figure 3 compares survey and computed tax rates over the distribution of reported income. The observations are grouped into ten equally sized bins of income. The mean tax rate is graphed at the mean income within the bin. The sample used for these figures was restricted to people who provided both ATR and MTR, such that the sample is the same in both figures, and respondents with income above $500,000 are removed to limit the mean of the binned income for the top bin.

Figure 3a shows that survey ATR is systematically larger than the computed ATR across the income distribution. To better understand this result, it is worth noting that computed ATR tends to overestimate the true ATR. By ignoring tax credits and assuming all income is taxed as wage or salary income, the tax liability computation is an upper bound on true tax liability at a given TI. This is because tax credits reduce tax liability and tax-preferred investments are taxed at lower rates than wages and salary income. When tax liability is an upper bound, the computed ATR tends to overestimate true ATR. So, if anything, I underestimate the difference between survey and true ATR. This provides strong evidence that respondents overestimate tax liability when answering the ATR question.

Figure 3b compares survey and computed MTRs across values of gross income. The mean survey MTR tracks the mean computed MTR. While the survey MTR is accurate on average, people at lower levels of income overestimate their MTR relative to the computed rates, whereas people at higher incomes underestimate MTR. In other words, the perceived tax schedule is flatter than the actual tax schedule, which can be interpreted as underestimation of tax rate progressivity.

## Discussion and Conclusion

The survey data analyzed in this article reveal systematic errors in perceptions of income tax rates. When asked about the MTRs for households in the top tax bracket, respondents underestimate the MTR on wages and salary income, overestimate the MTR on dividend income, and therefore significantly underestimate the currently tax-advantaged status of dividend income. People overestimate their tax burden (as reflected in their perceived ATR). And while the reported MTRs are accurate at the mean, respondents with lower income overestimate their MTR and those with higher income underestimate MTR. These results together suggest that people underestimate the degree of progressivity and the amount of differentiation across levels and types of income embedded in the tax code.

My results affirm and also contrast with previous evidence about perceptions of tax rates. Older studies of tax perceptions using survey or interview data find mixed results across time and different countries. Some studies find taxpayers slightly overestimate their tax liability (Wagstaff 1965; Kapteyan and Ypma 2007), while others find they slightly underestimate it (Enrick 1963, 1964).^7^ Some studies find that taxpayers overestimate their marginal tax rate (Brown 1968), while others find taxpayers underestimate it (Lewis 1978; Fujii and Hawley 1988). There is also previous evidence of lower income households overestimating and higher income households underestimating tax liabilities (Wagstaff 1965; Auld 1979). Finally, the evidence that people underestimate tax progressivity is consistent with Slemrod (2006), who finds that, in a question directly asking about the relative tax burden of middle- and high-income families, 50 percent of respondents think that middle-income families pay a higher percentage of income in taxes than do high-income families.

Tax misperceptions can be understood in the context of bounded rationality. There is public information about tax rates yet imperfect knowledge. One explanation is that taxpayers are rationally inattentive. The idea is that when information is costly to acquire, it may be rational for agents to act on incomplete information. In fact, the complexity of the US tax code makes it all but impossible to fully understand. Given finite cognitive resources, it seems rational to ignore policies that do not impact one's own immediate decisions and then attentive when making decisions. I find that some people know their own tax rates and understand the tax rate structure, while many others do not. This is consistent with Hoopes, Reck, and Slemrod (2015), who present convincing evidence that taxpayers are neither fully informed nor completely ignorant and at least some taxpayers employ rational *attention* to tax policies.

These results should be interpreted in light of issues of sample selection. In particular, evidence of systematic misperceptions in the CogEcon sample suggests this is an important phenomenon overall. The CogEcon sampling frame includes Americans aged 50 and older. Moreover, respondents have higher cognitive ability and income than the typical person their age. Older, better educated, and more intelligent households are likely more knowledgeable about tax rates than the population overall.

Caveats notwithstanding, this new evidence of how people perceive tax rates has important implications for research and policy. First, the overall response to tax policy changes is likely dampened by imprecise perceptions. This suggests that efficiency costs of taxation are lower than under correct perceptions. Second, however, the presence of tax rate misperception suggests that most empirical research about taxes does not observe the correct “treatment” to which people respond. Without measuring perceptions, we cannot readily distinguish between heterogeneous preferences and heterogeneous perceptions.

Finally, the findings also have important implications for understanding political debates about tax rates. As Sheffrin (1994) argues, ignorance about current tax rates might distort stated preferences about tax reform. Indeed, Slemrod (2006) has found “ … that the sense of unfairness about the current system has two distinct sources: the belief that taxes are generally too high, and the belief that high-income people are not paying their fair share of the overall tax burden” (p. 4). The results in this article suggest that voters might think the existing tax system is less fair than it is. The evidence suggests that increasing knowledge of the actual tax rates might make people less concerned about the top rate on wage/salary income and more concerned about the tax preference for investment income.

## Figures and Tables

**Table 1:** Sample versus Population Characteristics.

Variable	CogEcon	US population
Male (percent)	43.0	49.2
White alone, not Hispanic or Latino (percent)	90.0	63.7
Age 65+ (percent)	53.6	13.0
HS graduate or higher (percent)^a^	96.0	86.0
Bachelor's degree or higher (percent)^a^	52.8	28.8
Employed during 2010 (percent)^b^	50.2	58.5
Homeownership rate (percent)	86.7	64.9
Median household income^c^	65,638	53,046
Observations	748	308,745,538

*Source:* CogEcon 2011; U.S. Census Bureau QuickFacts, quickfacts.census.gov; U.S. Census Bureau. 2011. *Statistical Abstract of the United States:* 2012, 131st ed. Washington, DC. http://www.census.gov/compendia/statab/

*Note:* Unless stated otherwise, the U.S. population estimates are for 2010 and use Census QuickFacts. CogEcon = Cognitive Economics Study; HS = high school.

a For the U.S. population, this is the percent of persons twenty-five plus, 2009–2013.

b This comes from the 2012 Statistical Abstract, table 586; it is based on the civilian noninstitutional population, age sixteen plus.

c In CogEcon this is for 2010, the population estimates are for 2009–2013.

**Table 2:** Summary Statistics for Tax Rates.

Variable	Mean	Twenty-fifth percentile	Fiftieth percentile	Seventy-fifth percentile	*SD*	Observations	Missing
Top MTR in 2010 (wages)	27.4	18.0	30.0	35.0	1 1.9	531	217 (29.0%)
Top MTR in 2010 (dividends)	20	15.0	15.0	27.0	12.7	520	228 (30.5%)
E (top MTR) in 2014 (wages)	30.1	20.0	30.0	38.0	14.1	535	213 (28.5%)
E(top MTR) in 2014 (dividends)	22.6	15.0	20.0	30.0	13.3	526	222 (29.7%)
Own ATR in 2010	15.4	7.5	15.0	23.0	11.7	632	116 (15.5%)
Own MTR in 2010	16.1	10.0	15.0	25.0	11.3	555	193 (25.8%)
Expected own MTR in 2014	17.7	10.0	18.0	25.0	11.6	564	184 (24.6%)

*Source:* CogEcon 2011.

*Note:* The first four rows show survey responses about respondents' perceptions of the top tax rates in the tax code, the first two about the top rates in 2010, and the next two about the expected top rates in 2014. The final three rows show the respondents' beliefs about their own tax rates. MTR = marginal tax rate; ATR = average tax rate.

**Table 3:** Are Dividends or Wages Tax-advantaged at High Income?

Responses	Frequency	Percentage
Top div. MTR > top wage MTR	59	7.89
Top div. MTR = top wage MTR	162	21.66
Top div. MTR < top wage MTR	297	39.71
Missing	230	30.75
Total	748	100

*Source:* CogEcon 2011.

*Note:* This table summarizes the responses to survey questions about the advantages of dividends or wages for the entire sample of 748 respondents. MTR = marginal tax rate.

**Table 4:** Categories of ATR and MTR Responses.

	All respondents		Filed tax return	
	
	Frequency	Percentage	Frequency	Percentage
Skipped MTR & ATR	115	15.4	92	13.6
MTR, skipped ATR	1	0.1	1	0.2
ATR, skipped MTR	78	10.4	75	11.1
ATR > MTR	107	14.3	101	14.9
ATR = MTR	219	29.3	214	31.5
ATR < MTR	166	22.2	162	23.9
ATR = MTR = 0	62	8.3	34	5.0
Total	748	100	679	100

*Source:* CogEcon 2011.

*Note:* This table summarizes the responses to the ATR and MTR survey questions for the entire sample of 748 respondents and also for the 679 respondents (90.8 percent) who filed a tax return for 2010. MTR = marginal tax rate; ATR = average tax rate.

**Table 5:** Is MTR a MTR Statutory Rate?

Response category	Yes	No	Zero	Observations
ATR > MTR	42.1	41.1	16.8	107
ATR = MTR	49.8	50.2	0.0	219
ATR < MTR	66.9	33.1	0.0	166
ATR = MTR = 0	0.0	0.0	100.0	62
Total	47.8	37.7	14.4	554

*Source:* CogEcon 2011.

*Note:* This table shows the fraction of respondents who reported a nonzero statutory MTR (10, 15, 25, 28, 33, or 35 percent), broken down into categories based on responses to ATR and MTR. MTR = marginal tax rate; ATR = average tax rate.

**Table 6:** Taxable Income Thresholds by Tax Bracket and Filing Status (2010).

MTR	Single filers	Married filing jointly
10	0	0
15	8,375	16,750
25	34,000	68,000
28	82,400	137,300
33	171,850	209,250
35	373,650	373,650

*Source:* IRS Tax Code.

*Note:* This table shows the lower end of the taxable income thresholds for each tax bracket. The tax brackets are associated with marginal tax rates (MTR), and the columns distinguish between single and married tax filing status.

## Footnotes

1 Along these lines, Chetty, Friedman, and Saez (2013) exploit heterogeneity in knowledge to estimate behavioral responses to the earned income tax credit. However, they use observed neighborhood-level behavior as a proxy for individual knowledge, making it difficult to disentangle preferences and knowledge.

2 The Cognitive Economics Study (CogEcon) is supported by National Institute on Aging program project P01-AG026571, “Behavior on Surveys and in the Economy Using HRS,” Robert J. Willis, PI. CogEcon is an innovative new study launched in 2008 with a nationally representative sample of respondents aged fifty-one and over, including the respondents’ partners, regardless of their age. The first two waves, CogEcon 2008 and CogEcon 2009, collected information about income, work, assets and debts, financial sophistication, and use of financial advice, among other topics. CogEcon 2011 repeated many questions from the prior waves and included new questions specifically aimed at measuring tax knowledge and tax-related behaviors. There was an internet mode and a mail mode, with questions designed to be as similar as possible across mode. Households with Internet access were invited to the web version, and this ended up being two-thirds of respondents. More information about CogEcon is available online (http://cogecon.isr.umich.edu/survey/) and in the data documentation (Fisher et al. 2011; Gideon, Koch, and McFall 2013). CogUSA is part of the Unified Studies of Cognition led by cognitive psychologist Jack McArdle at the University of Southern California. All respondents who completed the first waves of CogUSA, and were not otherwise involved with the Health and Retirement Study, were invited to complete the first CogEcon survey. More information about CogUSA is available at cogusc.usc.edu.

3 Pilot testing revealed that respondents better understood questions explicitly asking for the marginal tax rate than questions asking for the tax rate on the “last $100 of income.” Preliminary versions of the survey instruments were tested on two groups. First, they were tested by people who were in the demographic of the CogEcon respondents—Americans aged fifty and older, who are slightly better educated and with higher income than the age demographic at large. Second, they were tested as part of the standard Survey Research Operations procedure for testing survey instruments. After testing both versions, we learned that people would sometimes say zero because they were thinking of the paper tax table and whether or not the additional money would actually move them from one cell in the tax table to another cell. Other testers mentioned that US$100 is small and that the tax can’t be much.

4 Prior to 2003, dividends were taxed as ordinary income, but the Jobs and Growth Tax Relief Reconciliation Act of 2003 (JGTRRA) reduced the tax rate on qualified dividends to the long-term capital gains rate of 15 percent.

5 If married respondents file separately, then I will underestimate their true MTR and therefore overestimate the difference between perceived and true MTR; the opposite will be true for singles, for whom I will overestimate their MTR and underestimate their bias.

6 This procedure ignores some important institutional details. First, income from some investments gets taxed according to a different rate schedule. Prior to 2003, dividends were taxed as ordinary income, but JGTRRA reduced the top tax rate on qualified dividends to the long-term capital gains rate of 15 percent. Second, I ignore the possibility that respondents are subject to the Alternative Minimum Tax.

7 Kapteyn and Ypma (2007) do not focus on tax perceptions, yet have results that address the question of self-reported versus true tax liability. They use data from Sweden and find that people overreport their income tax liability relative to the tax liability in administrative records. The Swedish administrative data come from Longitudinal Individual Data for Sweden, and the survey data are from Survey of Health, Ageing, and Retirement in Europe.

## Appendix: Survey Instrument

### Instructions for the following questions

These questions focus on the current and future federal income tax rates, both in general and for you personally.

The *marginal tax* rate is the tax rate on the last dollars earned. For example, if a household's income tax bracket has a marginal tax rate of 15%, then a household owes an extra $15 of taxes when it earns an extra $100.

Answer each question with a percentage between 0 and 100. Please provide your best estimate of the marginal tax rate even if you are not sure.

These questions are about *federal income taxes only*; please do not include state or local taxes or payroll taxes for social security and medicare.

G6. For a household in the *highest tax bracket* in *2010*:

The marginal tax rate on *wage and salary income* was ____ %, and the marginal tax rate on *dividend income* was ____ %.

G7. Tax rates may change in the future. I think that for a household in the highest tax bracket in *2014*:

The marginal tax rate on wage and salary income will be ______ %, and the marginal tax rate on dividend income will be ____ %.

We now want to ask you about your household's federal taxes. Please use the same definitions of federal income tax and marginal tax rate as on the previous page.

G8. Please think about your household's income in 2010 and the amount of federal income tax you paid, if any.

Approximately what percentage of your household income did you pay in federal income taxes in 2010? ____ %.

G9. Now, we want to ask about *your households' marginal income tax rate*. Please think about your household's federal income tax bracket and the tax rate on your last dollars of earnings.

*In 2010*, my household's marginal tax rate was ____ %.

G10. Suppose that in *2014* your household receives the same income you had in 2010. However, the federal income tax schedule might change.

I expect that my household's marginal tax rate in 2014 would be ____ %.
